# Clinical and biomechanical outcomes following patellar tendon repair with suture tape augmentation

**DOI:** 10.1007/s00590-023-03572-4

**Published:** 2023-05-26

**Authors:** Maximilian Hinz, Stephanie Geyer, Felix Winden, Alexander Braunsperger, Florian Kreuzpointner, Markus Irger, Andreas B. Imhoff, Julian Mehl

**Affiliations:** 1 Department of Sports Orthopaedics, Technical University of Munich, Ismaninger Straße 22, 81675 Munich, Germany; 2 Department of Sport and Health Sciences, Prevention Center, Technical University of Munich, Munich, Germany

**Keywords:** Quadriceps, Sports Injury, Winter sports, Ball sports, Internal brace, Return to sports, Acute, Chronic

## Abstract

**Purpose:**

Patellar tendon ruptures (PTR) occur predominantly in middle-aged patients following indirect trauma. The aim of this study was to quantify the short-term results using a suture tape augmentation technique for the repair of PTR.

**Methods:**

All consecutive patients with acute (< 6 weeks) PTR who underwent suture tape augmentation between 03/2014 and 11/2019 at a single institution with a minimum follow-up of 12 months were retrospectively evaluated. Outcome measures included Visual Analog Scale (VAS) for pain, Tegner Activity Scale (TAS) and return to sport rates, Lysholm score, International Knee Documentation Committee subjective knee form (IKDC) as well as Knee Injury and Osteoarthritis Outcome Score (KOOS). Additionally, a standardized clinical examination and an isometric strength evaluation of knee extension and flexion were performed. It was hypothesized that high return to sport rates and good functional outcome would be observed and that the majority of patients would not present with a severe (> 20%) knee extension strength deficit when compared to the contralateral side.

**Results:**

A total of 7 patients (mean age 37.0 ± SD 13.5 years; 6 male/1 female) were available for final assessment at a median follow-up of 17.0 (25–75% IQR 16.0–77.0) months. Three injuries occurred during ball sports, two injuries occurred during winter sports, and one injury each occurred during a motorcycling and skateboarding accident. The average time between trauma and surgery was 4.7 ± 2.6 days. At follow-up, patients reported little pain (VAS: 0 [0–0.4]). Return to sport was possible for all patients 8.9 ± 4.0 months postoperatively at a high level (TAS: 7.0 [6.0–7.0]). Five patients (71.4%) returned to the preinjury level of play, and 2 (28.6%) did not return to the preinjury level of play. Patient-reported outcome measures were moderate to good (Lysholm score: 80.4 ± 14.5; IKDC: 84.2 ± 10.6; KOOS subscales: pain 95.6 ± 6.0, symptoms 81.1 [64.9–89.1], activities of daily living 98.5 [94.1–100], sport and recreation function 82.9 ± 14.1 and knee-related quality of life 75.9 ± 16.3). All patients were very satisfied (57.1%) or satisfied (42.9%) with the postoperative result. No postoperative complications were reported. Strength measurements revealed a severe knee extension deficit in 3 patients (42.9%), but no significant deficit of isometric knee extension or flexion strength in comparison with the contralateral side was observed overall (*p* > 0.05).

**Conclusion:**

Suture tape augmentation in acute PTR repair leads to good functional outcome without major complications. Although a severe knee extension strength deficit may occur in some patients postoperatively, an excellent return to sports rate and high patient satisfaction can be expected nonetheless.

**Level of evidence:**

Retrospective cohort study; III.

## Introduction

Patellar tendon ruptures (PTR) are rare injuries with a reported incidence of 0.68 per 100,000 person-years [1] that usually occur in middle-aged patients due to abrupt quadriceps contraction with moderate knee flexion (e.g., sprinting or avoiding a fall) [2].

Complete PTR are typically treated surgically; in mid-substance tears an end-to-end suture may be performed, whereas in avulsion injuries from the tip of the patella the tendon may be reinserted using transosseous sutures or suture anchors [2]. However, rerupture rates of up to 7.5% are reported [3]. In an effort to achieve the most favorable clinical result, many of the proposed refixation techniques involve a non-metal augmentation to protect the repair and allow for an early rehabilitation [2, 4–6]. The replacement of metal wire augmentations with sutures has been proposed to reduce the risk regarding surgical re-intervention for implant removal [7]. Through a primary repair of acute PTR with an augmentation, superior clinical results with a failure rate of 2% may be achieved [5]. It has also been shown in cadaveric studies that suture augmentation significantly decreases gap formation under cyclic loading and increases the maximum load to failure, potentially allowing for earlier mobilization [8–12].

Furthermore, Besa et al. [13] investigated the biomechanical properties of an isolated suture frame without end-to-end sutures that may interfere in the biology of the tendon repair. They reported a significantly smaller gap formation under cyclic loading compared to when a Krackow fixation was performed. Augmentation techniques using high-strength suture tapes have previously been shown to be effective in treating various knee, shoulder, elbow and ankle pathologies [14–20].

However, there is lacking evidence regarding the functional and strength outcome following suture tape augmentation techniques for the repair of acute PTR. Therefore, besides clinical outcomes, the present study sought to quantify functional and strength results via an isokinetic dynamometer.

It was hypothesized that, following suture tape augmentation for the repair of acute PTR, high return to sport rates and good functional outcome would be observed and that the majority of patients would not present with a severe knee extension strength deficit (defined as > 20% in consonance with current literature on knee extensor tendon ruptures [21]) when compared to the contralateral side.

## Material and methods

### Study cohort

**Fig. 1:**
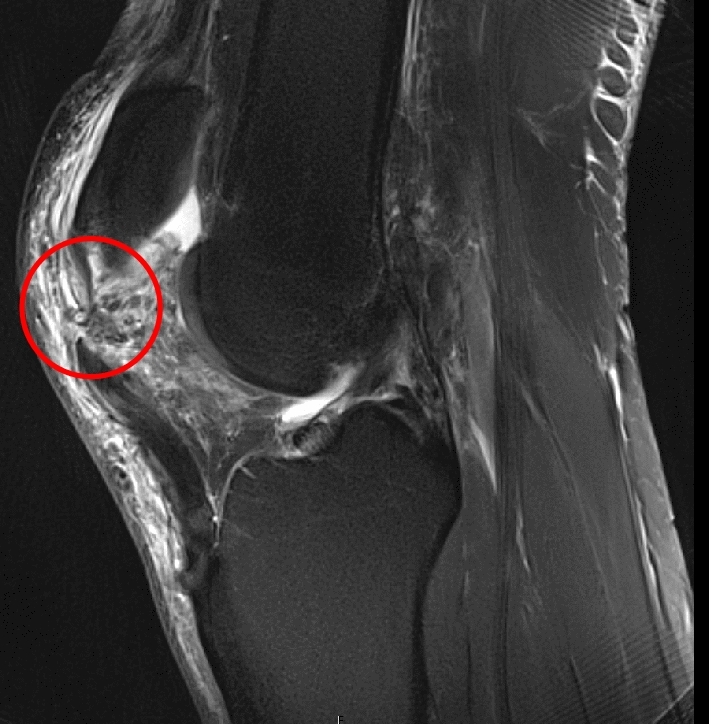
Intermediate-weighted (proton density) sagittal magnetic resonance imaging showing proximal avulsion of the patellar tendon (red circle)

Patients who underwent open surgical repair of isolated PTR with suture tape augmentation between 03/2014 and 11/2019 were included for retrospective review at a minimum follow-up of 12 months. Patients were included if an acute (< 6 weeks since trauma) PTR had been confirmed by both clinical examination and preoperative magnetic resonance imaging (Fig. 1). Due to the debilitating nature of the injury, early operative intervention was performed. Exclusion criteria were chronic injuries (> 6 weeks since initial trauma) and recurrent or contralateral PTR injuries.

### Surgical technique

**Fig. 2:**
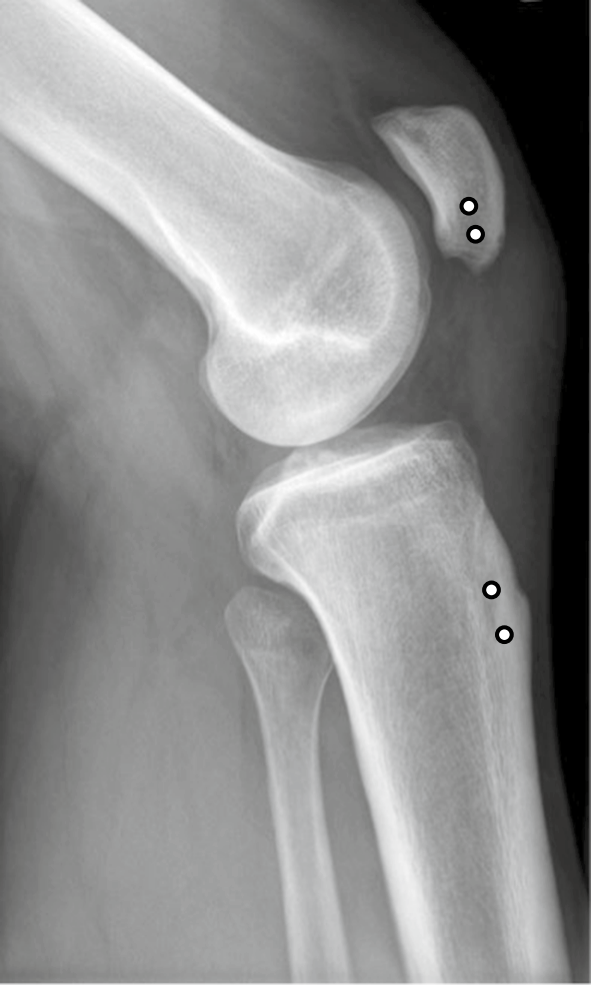
Preoperative lateral radiograph showing a patella alta in a patient with a patellar tendon rupture. The recommended tunnel position for the suture augmentation is marked (white circle with black border)

All patients were operated on at a single institution by experienced orthopedic sports medicine surgeons. A detailed description of the authors’ preferred operative technique was previously described [22]. In brief, a longitudinal anterior skin incision was made from 2 cm proximal to the distal patellar pole to the tibial tuberosity. The enthesis of the patellar tendon was visualized, and scar tissue was debrided. The suture tape augmentation was performed by drilling two bone tunnels horizontally each through the distal patella (open) and tibial tuberosity (percutaneously) using a cannulated 2.4 mm drill with a minimum distance of 5 mm between each tibial and patellar tunnel (Fig. 2). Two separate braided high-strength suture tapes (FiberTapes®, Arthrex Inc., Naples, USA) were shuttled through the proximal and distal bone tunnels, surrounding the tendon in an “X”- and “O”-type configuration. After reestablishing the correct patellar height under fluoroscopic control with 90° of knee flexion and in correlation to the uninjured side, both suture tapes were tied down—as previously described [22]. In cases of proximal tendon avulsion, the tendon insertion—the inferior margin of the patella—was debrided and the anatomical footprint was decorticated to facilitate healing. The tendon reinsertion was then performed with two double-loaded 5.5 mm titanium suture anchors (Corkscrew®, Arthrex, Naples, USA) under fluoroscopic control in 90° of knee flexion. In these cases, the suture tape cerclages were added in the same manner to augment the suture anchor reinsertion of the patellar tendon. In selected cases with a proximal tendon stump on the patellar side, the entheses were adapted using absorbable sutures instead. Following that, the repair augmentation was performed in the same manner. Finally, the wound was irrigated and closed.

### Postoperative rehabilitation

After surgery, the affected leg was secured in a knee brace (M.4 X-lock®, medi GmbH & Co. KG, Bayreuth, Germany) for six weeks with knee flexion limited to 30°, 60° and 90° for 2 successive weeks, respectively. During this time, weight bearing was restricted to ≤ 20 kg in full knee extension only. After a follow-up examination 6 weeks postoperatively, patients were encouraged to steadily increase the weight put on the operated leg until full weight bearing was achieved. Physiotherapy started on the first postoperative day with passive flexion to 30° (1–2 week), 60° (3–4 week) and 90° (5–6 week). Additionally, isometric quadriceps exercises in supine position with the knee in full extension were encouraged during the first six postoperative weeks. Active knee extension exercises were started from the seventh postoperative week. Patients received physiotherapy treatments 2–3 times per week.

### Outcome parameters

**Fig. 3:**
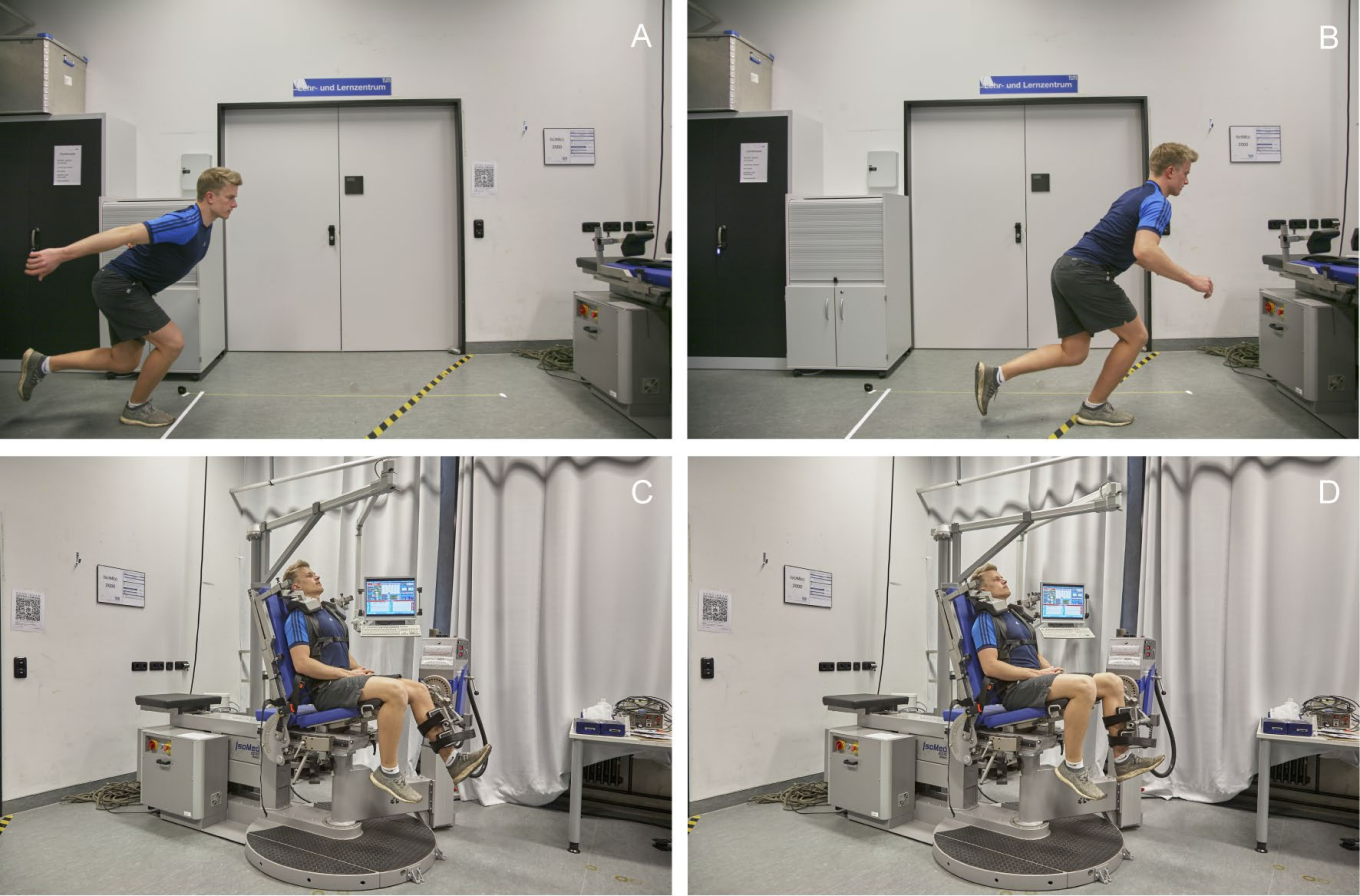
Postoperative lower extremity function was assessed by measuring the single-leg hop for distance (**A** and **B**). Isometric knee extension (**C**) and knee flexion (**D**) strength was measured using an isokinetic dynamometer (IsoMed® 2000, D&R Ferstl GmbH, Hemau, Germany) at 60° of flexion. Images **A**, **C** and **D** were used in two prior studies [26, 27], and image **B** was used in one prior study [26]

At a single follow-up appointment, patient-reported outcome measures (Visual Analog Scale [VAS] for pain, Tegner Activity Scale [TAS], Lysholm score, International Knee Documentation Committee subjective knee form [IKDC], Knee Injury and Osteoarthritis Outcome Score [KOOS]) were collected to quantify subjective knee function and to assess postoperative pain as well as sports participation. Furthermore, data on return to work and sport rates, the level of satisfaction with the postoperative result, and information on postoperative complications, with a special focus on injury recurrence, were collected. Objectively, range of motion (ROM) of the knee, thigh circumference (as an average of two measures: 10 and 15 cm superior to the joint line of the knee), and heel-to-buttock distance were measured to detect potential loss of muscle size or reduced flexibility. Lower extremity function was assessed using the single-leg hop (SLH) for distance (Fig. 3a, b), which has been frequently used as a postoperative functional performance test following lower extremity injuries [23–28]. Finally, isometric strength was evaluated using an isokinetic dynamometer (IsoMed® 2000, D&R Ferstl GmbH, Hemau, Germany). This was achieved by measuring maximum voluntary isometric contraction (MVIC), as peak torque in Newton meters (N x m), unilaterally in single-joint knee extension and flexion. Following a standardized warm-up session, which was intended to activate the cardiovascular system, patients were seated in an upright position and secured by shoulder pads and hip and shoulder belts. The rotational dynamometer was then calibrated to the body’s dimensions. Two adjustable straps secured the leg at the pad of the lever arm in 60° of knee flexion. Following a mock session with the test equipment and set-up, knee extension strength was measured by asking the patients to extend the knee using maximum quadriceps contraction for 5 s (Fig. 3c). Knee flexion strength was measured in the same manner by asking the patients to pull against the measuring pad with maximum hamstring muscle contraction for the same amount of time (Fig. 3d). Maximum voluntary isometric contraction was measured three times for each muscle group with three minutes of rest in between each repetition. For each muscle group, the highest maximum isometric torque was used for data analysis. The starting leg and order in which each muscle group was tested was randomized [29]. Strong verbal encouragement was provided during testing to ensure maximum effort [30]. Using peak torque measures of knee flexion and extension, the hamstring-to-quadriceps ratio $\left( {{\text{H}}:{\text{Q}} = \frac{{\text{peak hamstrings torque}}}{{\text{peak quadriceps torque}}} \times 100\% } \right)$ was calculated [31]. Finally, the limb symmetry index (LSI) was calculated using the measurements of the injured and uninjured limb $\left( {{\text{LSI}} = \frac{{\text{injured leg}}}{{\text{uninjured leg}}} \times 100} \right)$ [32]. The study was approved by the ethics committee of the Technical University of Munich (reference: 317/20 S). It was conducted according to the Declaration of Helsinki and all patients signed informed consent forms.

### Statistical analysis

Data were analyzed using SPSS 26.0 (IBM-SPSS, New York, USA). Categorical variables are presented in sums and percentages. Normal distribution of the collected continuous variables was assessed by the Shapiro–Wilk test and graphically confirmed. Accordingly, continuous variables are presented either as mean and standard deviation or as median and 25–75% interquartile range. For group comparison of continuous variables, the paired *t* test was applied for normally distributed data. Statistical significance was set at a *p* value of < 0.05.

Due to the rarity of the injury, all eligible patients were included and a power analysis was not carried out.

## Results

### Demographics

A total of 14 PTR reconstructions were performed in the aforementioned time frame. Four patients also suffered concomitant ligamentous knee injuries and two patients had secondary contralateral PTR and were therefore excluded. Of the remaining 8 patients that were eligible for inclusion, 7 (87.5%) participated in the physical examination and strength assessment. One patient did not consent to participate and was therefore excluded. Of those 7 PTR, 3 occurred during ball sports (1 × basketball, 1 × handball, 1 × soccer), 2 occurred during winter sports (1 × alpine skiing, 1 × snowboarding), and one each occurred during a motorcycling and skateboarding accident. The median follow-up was 17.0 (16.0–77.0) months. The patients’ demographics are shown in Table 1.

**Table 1:** Patients’ demographics

	Number of patients
Number of patients (*N*)	7
Sex (male/female)	6/1 (85.7% male)
BMI (kg/m^2^)	27.6 ± 4.1
Injured side (left side/right side)	5/2 (71.4% left side)
Age at time of surgery (years)	37.0 ± 13.5
Time from trauma to surgery (days)	4.7 ± 2.6
Follow-up (months)	17.0 (16.0–77.0)

Normally distributed continuous variables are shown as mean ± standard deviation. Non-normally distributed continuous variables are shown as median (25–75% interquartile range). Categorical variables are shown as percentages

### Patient-reported outcome measures

At follow-up, the patient-reported outcome by means of Lysholm score, IKDC and KOOS subscales (pain, sport and recreation function, knee-related quality of life) as well as median VAS, TAS and KOOS subscales (symptoms, activities of daily living) was moderate to good (Table 2). All patients returned to sports at a mean of 8.9 ± 4.0 months. Five patients (71.4%) returned to the preinjury level of play, of which 3 reported minor limitations and 2 (28.6%) did not return to the preinjury level of play. Return to work was achieved by all patients as well. Furthermore, all patients were very satisfied (*N* = 4, 57.1%) or satisfied (*N* = 3, 42.9%) with the postoperative results. No postoperative complications or revision surgeries were reported.

**Table 2:** Patient-reported outcome measures

Patient-reported outcome measures	Results
VAS for pain	0 (0–0.4)
Lysholm score	80.4 ± 14.5
TAS	7.0 (6.0–7.0)
IKDC	84.2 ± 10.6
*KOOS*	
Pain	95.6 ± 6.0
Symptoms	81.1 (64.9–89.1)
Activities of daily living	98.5 (94.1–100)
Sport and recreation function	82.9 ± 14.1
Knee-related quality of life	75.9 ± 16.3

Normally distributed continuous variables are shown as mean ± standard deviation. Non-normally distributed continuous variables are shown as median (25–75% interquartile range)

*IKDC* International Knee Documentation Committee subjective knee form; *KOOS* Knee Injury and Osteoarthritis Outcome Score; *TAS* Tegner Activity Scale; *VAS* Visual Analog Scale

### Functional outcome

Knee ROM, heel-to-buttock distance, and thigh circumference were comparable between the operated leg and the contralateral leg. Furthermore, no significant strength or functional impediment of the operated leg was detected using MVIC of knee flexion and extension, and the SLH for distance. However, an MVIC_knee extension_ deficit > 20% was observed in three patients (42.9%). The H:Q was similar between both legs, and LSI were overall favorable (Table 3).

**Table 3:** Results of the strength and functional assessment

	Operated leg	Contralateral leg	*p* value
ROM knee flexion (degrees)	129.3 ± 13.0	130.0 ± 22.2	.912
ROM knee extension (degrees)	+ 6.4 ± 3.8	+ 7.1 ± 4.9	.805
Heel-to-buttock distance (cm)	15.0 ± 8.2	14.7 ± 9.3	.934
Thigh circumference (cm)	46.6 ± 3.9	48.5 ± 5.4	.068
SLH distance (cm)	117.0 ± 34.0	136.3 ± 23.0	.138
MVIC_knee extension_ (N*m)	190.0 ± 55.1	218.4 ± 23.2	.259
MCIV_knee flexion_ (N*m)	110.6 ± 26.0	102.7 ± 9.3	.845
H:Q (%)	55.1 ± 13.0	47.1 ± 1.8	.156
*LSI*			
Knee extension	88.0 ± 27.3		
Knee flexion	89.8 (80.1–100.2)		

Normally distributed continuous variables are shown as mean ± standard deviation. Non-normally distributed continuous variables are shown as median (25–75% interquartile range)

*H:Q* Hamstring-to-quad ratio; *LSI* Limb symmetry index; *MVIC* Maximum voluntary isometric contraction; *ROM* Range of motion; *SLH* Single-leg hop

## Discussion

The most important finding of the study was that suture tape augmentation for repair of acute PTR leads to moderate to good patient-reported outcomes and enables a high postoperative sports activity. Additionally, the MCIV_knee extension_ was not significantly compromised compared to the contralateral leg with the majority of patients not showing a severe (> 20%) knee extension strength deficit at follow-up. The study population with an 85.7% male gender distribution and average age of 37.0 ± 13.5 years at time of surgery resembled a typical cohort suffering from PTR [2, 3, 33].

Various studies on knee, shoulder, elbow and ankle pathologies have shown that suture tape augmentation leads to an excellent outcome allowing for earlier mobilization and subsequent earlier return to activity due to protection of the primary repair [14–20].

To our knowledge, studies have not yet described the clinical outcome following an isolated suture augmentation of PTR, but have previously reported good to excellent results following metal-free augmentation of acute PTR reconstructions [2, 6, 33, 34]. West el al. [6] reported on 30 patellar tendon ruptures that were treated with transosseous tendon refixation and two additional high-strength sutures (Ethicon Inc., Johnson & Johnson, Somerville, USA) that served as “relaxing sutures” in order to enable early motion, weightbearing and brace-free ambulation. They reported good patient-reported outcomes without associated complications. Roudet et al. [2] reported the outcome following refixation of acute PTR in 38 cases, of which 36 were augmented by a hamstring graft, synthetic ligament or metal wire. They reported that both the short-term (mean 7.1 months) and long-term outcomes (mean 9.3 years) were satisfactory. Kasten et al. [34] compared two different augmentation techniques (22 patients with metal wire cerclage augmentation vs. 7 patients with polydioxanone [PDS] cerclage augmentation) in 29 cases of PTR reconstructed with end-to-end sutures. They reported that at a mean of 8.1 years postoperatively, no reruptures occurred and no extension lag was observed in patients that received PDS cerclage augmentation. Two patients, however, presented with extension lag in the group that underwent metal wire cerclage augmentation. Further, two patients (28.6%) that received PDS cerclage augmentation had a postoperative wound infection requiring revision surgery, which was not found in the present collective where the augmentation was performed using non-absorbable suture material. Beranger et al. [33] reported on 23 cases (20 patients) of acute PTR which were reconstructed with transosseous or end-to-end suture and augmented with a non-absorbable size 7 Terylene® suture (Peters Surgical, Boulogne-Billancourt, France). At a mean follow-up of 47.7 months, they reported a high patient satisfaction and good patient-reported outcome comparable to our study’s findings. In their collective, a worse prognosis was observed in patients aged > 40 and those with a body mass index (BMI) > 25. The vast majority of patients returned to sporting activities and their preinjury level of activity. In the present study, patients also reached a high sporting activity level postoperatively. Regarding the sporting activity level following patellar tendon repairs, a recent systematic review reported an overall rate of return to play of 88.9% with 80.8% returning to the same level of play, which is comparable to our study’s findings [35]. Of the 33 studies that reported on patellar tendon repairs, 4 studies reported on the postoperative TAS (mean weighted TAS: 4.5 (range, 1.7–7.1), which was, however, considerably lower than in our study (median TAS: 7.0 [6.0–7.0]).

Only few studies assessed postoperative quadriceps strength following reconstruction of PTR [6, 34]. Kasten et al. [34] evaluated the isokinetic quadriceps strength in patients that underwent wire cerclage or PDS augmentation following end-to-end suture. In the PDS cerclage-group, a relative strength of 83 ± 13% (at 60°/s) and 100 ± 16% (at 180°/s) was found with no significant difference compared to the metal wire cerclage-group. West et al. [6] assessed the strength levels of 23 patients that underwent either quadriceps or patellar tendon reconstruction which were augmented with a “relaxing suture”. Isokinetic quadriceps strength testing was performed 12 months postoperatively, which revealed a mean quadriceps strength deficit of 35% at slow speed (60°/s) and of 38.8% at high speed (240°/s). The high quadriceps strength discrepancy between the study from West et al. and the aforementioned study by Kasten et al. may be due to the varying follow-up (12 months vs. 8.1 years [range, 1–16 years]) and the consequently varying rehabilitation time. Furthermore, West et al. did not report separately between quadriceps and PTR which may limit the extrapolation of their findings. However, knee extension strength is known to be compromised significantly following quadriceps tendon reconstruction after acute quadriceps tendon tears [27].

Previous studies have reported implant-related complications using high-strength suture tapes. Heterotopic ossification and stiffness when treating elbow instability [15, 16] and a higher rate of peroneal nerve and tendon irritation when performing a Broström procedure have been described [20]. These complications, however, did not interfere with the continuing recommendation of suture tape augmentation in order to facilitate earlier mobilization and return to activity. Furthermore, they were not observed in the current study on PTR.

This study has several limitations that must be considered when interpreting the findings. First, although a high-strength suture augmentation was used in all patellar tendon reconstructions, in some cases additional repair techniques were used (suture anchor refixation or end-to-end suture), which limits the extrapolation of our data to isolated suture augmentations. Second, as this study only had a small sample size and relatively short follow-up, future studies should focus on the outcome in larger cohorts, especially regarding potential negative side effects of the non-absorbable suture material.

The findings of this study will contribute to a more informed discussion about the use of a suture augmentation in the treatment of acute PTR and the expected postoperative result.

## Conclusion

Suture tape augmentation in acute PTR reconstruction leads to good functional outcome without major complications. Although a severe knee extension strength deficit may occur postoperatively in some patients, an excellent return to sports rate and high patient satisfaction can be expected nonetheless.
